# The relationship between long non-coding gene *CASC21* polymorphisms and cervical cancer

**DOI:** 10.1080/15384047.2024.2322207

**Published:** 2024-03-11

**Authors:** Lili Han, Jing Liu, Mireayi Shataer, Chengyong Wu, Mayinuer Niyazi

**Affiliations:** Department of Gynecology, People’s Hospital of Xinjiang Uygur Autonomous Region, Urumchi, Xinjiang, China

**Keywords:** Cervical cancer, genetic polymorphism, *CASC21*

## Abstract

**Background:**

*CASC21* was reported to be a hotspot gene in cervical cancer. The relationship between *CASC21* genetic polymorphisms and cervical cancer has not been reported. Genetic factors influence the occurrence of cervical cancer. Thus, we explored the correlation between *CASC21* polymorphisms and cervical cancer.

**Methods:**

A total of 973 participants within 494 cervical cancer cases and 479 healthy controls were recruited. Five single nucleotide polymorphisms (SNPs) in the *CASC21* gene were genotyped using the Agena MassARRAY platform. Chi-squared test, logistic regression analysis, odds ratio (OR), multifactor dimensionality reduction (MDR), and 95% confidence interval (95%CI) were used for data analysis.

**Results:**

In the overall analysis, rs16902094 (*p* = .014, OR = 1.86, 95% CI = 1.12–3.08) and rs16902104 (*p* = .014, OR = 1.86, 95% CI = 1.12–3.09) had the risk-increasing correlation with the occurrence of cervical cancer. Stratification analysis showed that rs16902094 and rs16902104 were still associated with cervical cancer risk in the subgroups with age > 51, BMI < 24 kg/m^2^, smokers, and patients with cervical squamous cell carcinoma. MDR analysis displayed that rs16902094 (.49%) and rs16902104 (.52%) were the main influential attribution factor for cervical cancer risk.

**Conclusion:**

Our finding firstly determined that two *CASC21* SNPs (rs16902094, rs16902104) were associated with an increased risk of cervical cancer, which adds to our knowledge regarding the effect of *CASC21* on cervical carcinogenesis.

## 1. Introduction

Cervical cancer is one of the most important causes of female deaths worldwide.^1^ Cervical cancer has a high incidence (8%) compared to other developed countries with a 6.5% incidence rate globally and a low survival rate in China (59.8% Age-standardized 5-year relative survival).^1,2^ According to the Global Cancer Observatory 2018 database(https://gco.iarc.fr/), China accounts for a third of cervical cancer cases worldwide.^3^ The most common symptoms of cervical cancer are contact or irregular vaginal bleeding, or increased leucorrhea after menopause.^4^ The main risk factors for cervical cancer are age, virus infections, sexually transmittable infections, smoking, and other factors.^5^ It has been shown that the progression of cancer and the occurrence of tumors is related to the polymorphism of gene loci, including cervical cancer.^6^ *IL1R2* and *TNF* genetic polymorphisms increase the risk of cervical cancer in Uygur women in China.^7,8^ The polymorphism in the CCR5 promoter region can affect the occurrence of cervical cancer in the Chinese Han population.^9^ TP53 Codon 72 polymorphism was reported to be also associated with cervical cancer.^10^ However, the association of a large number of loci with the risk of cervical cancer has not yet been studied.

Cancer susceptibility 21 (*CASC21*) is a FOXP1-induced long non-coding RNA (lncRNAs) for cancer susceptibility, located on homo sapiens chromosome 8 (8q24.21). There are currently very few studies on it. Abnormal expression of cyclin-CDK is one of the hallmarks of cancer. *CASC21* is a hotspot gene for HPV integration in RNA samples of cervical cancer.^11^ These studies suggested that *CASC21* might play a key role in cervical cancer tumorigenesis. Mutations in long non-coding RNAs are closely associated with the development of cancer. Many studies have shown that lncRNA polymorphisms are closely related to the development of cervical cancer.^12–14^ Therefore, we speculate that *CASC21* polymorphisms may be related to the occurrence of cervical cancer. Previously, rs16902094 was associated with susceptibility to prostate cancer in several European populations.^15^ An association between rs13281615 and rs1562430 polymorphisms and breast cancer susceptibility was reported.^16,17^ However, no studies have been done on the relationship between *CASC21* and the occurrence of cervical cancer.

These five SNPs (rs16902094, rs16902104, rs13281615, rs1562430, and rs2392780) were selected based on the following: 1) minor allele frequency (MAF) > .05 in the Chinese Han population from 1000 Genomes Chinese Han Beijing population and dbSNP database; 2) Hardy-Weinberg equilibrium (HWE) > .05, and the call rate for genotyping > 99.5%; 3) the related literature of *CASC21* polymorphisms.^15–17^ In this study, the aim was to investigate the relationship between *CASC21* single nucleotide polymorphisms (SNPs) and the risk of cervical cancer in the Han population from northwest China.

## 2. Result

### 2.1. Study population

In this study, 494 patients with cervical cancers and 479 controls were enrolled. The basic information about the cases and controls was displayed in Table 1. The mean age of cases and controls was 51.65 ± 9.84 years and 51.54 ± 9.46 years, respectively. There were no differences in age (*p* = .860), body mass index (BMI, *p* = .192), smoking (*p* = .930), and drinking (*p* = .674) between the two groups. In the case group, there were 197 cases (39.9%) of stage I-II, 196 cases (39.7%) of stage III-IV, and 101 cases (20.4%) of deletion. Of the 494 patients, 171 (34.6%) had squamous cell carcinoma.

**Table 1.** The information of all participants.

Variable		Cases	Control	*p*
Total		494	479	
Age, years		51.65 ± 9.84	51.54 ± 9.46	0.860
	>51	252 (51.0%)	247 (51.6%)	
	≤51	242 (49.0%)	232 (48.4%)	
BMI, kg/m^2^		23.46 ± 3.58	23.81 ± 4.68	0.192
	≥24	220 (44.5%)	191 (39.9%)	
	<24	274 (55.5%)	288 (60.1%)	
Smoking	Yes	242 (49.0%)	236 (49.3%)	0.930
	No	252 (51.0%)	243 (50.7%)	
Drinking	Yes	246 (49.8%)	245 (51.1%)	0.674
	No	248 (50.2%)	234 (48.9%)	
Clinical staging	I/II	197 (39.9%)		
	III/IV	196 (39.7%)		
	Missing	101 (20.4%)		
Tumor types	Squamous cell carcinoma	171 (34.6%)		
	Missing	323 (65.4%)		

*p* < .05 indicates statistical significance.

### 2.2. Association between SNPs in *CASC21* and cervical cancer risk

The allele, MAF, and other information of *CASC21* polymorphisms (rs16902094, rs16902104, rs13281615, rs1562430, and rs2392780) were shown in Table 2. All SNPs were consistent with HWE. The results of genotyping displayed that the genotyping success rate of each SNP was > 99.8%. The allele frequencies of rs16902094-G and rs16902104-T in the case group (.267 and .265) were higher than that in the control group (.225 and .227), and rs16902094-G (*p* = .033, Odd ratio (OR) = 1.25, 95% confidence interval (CI) = 1.02–1.54) and rs16902104-T (*p* = .048, OR = 1.23, 95% CI = 1.00–1.52) had the risk-increasing correlation with the occurrence of cervical cancer. HaploReg database displayed that these polymorphisms might be related to promoter/enhancer histone marks, DNAse, motifs changed, NHGRI/EBI GWAS hits, and selected eQTL hits.

**Table 2.** Basic information and allele frequencies of the five selected SNPs in *CASC21.*

SNP_ID	Chromosome	Position	Allele	MAF		HWE *p*-value	Call rate	OR (95% CI)	*p*	HaploReg
				Case	Control					
rs16902094	8q24	127308101	A/G	0.267	0.225	0.896	100.0%	**1.25 (1.02–1.54)**	**0.033**	Promoter histone marks, Enhancer histone marks, DNAse, Motifs changed, NHGRI/EBI GWAS hits
rs16902104	8q24	127328663	C/T	0.265	0.227	0.897	100.0%	**1.23 (1.00–1.52)**	**0.048**	Enhancer histone marks, DNAse, Motifs changed, Selected eQTL hits
rs13281615	8q24	127343372	G/A	0.487	0.477	0.099	99.9%	1.04 (.87–1.24)	0.664	Enhancer histone marks, Motifs changed, NHGRI/EBI GWAS hits
rs1562430	8q24	127375606	T/C	0.180	0.175	0.874	99.8%	1.03 (.82–1.31)	0.781	Enhancer histone marks, DNAse, NHGRI/EBI GWAS hits
rs2392780	8q24	127375779	A/G	0.261	0.264	0.347	100.0%	0.98 (.80–1.21)	0.882	Enhancer histone marks, DNAse, Motifs changed, Selected eQTL hits

MAF: minor allele frequency; HWE: Hardy-Weinberg equilibrium; SNP: Single nucleotide polymorphism; OR: Odds ratio; 95% CI: 95% confidence interval.

Bold *p* < .05 indicates statistical significance.

### 2.3. Associations between genotype frequencies and cervical cancer

The association of selected SNPs with the risk of cervical cancer was analyzed by four genotype models (Table 3). Rs16902094 in *CASC21* contributed to the increased risk of cervical cancer in the co-dominant (*p* = .042, OR = 1.91, 95% CI = 1.14–3.20), recessive (*p* = .014, OR = 1.86, 95% CI = 1.12–3.08), and log-additive (*p* = .039, OR = 1.24, 95% CI = 1.01–1.51) models. Rs16902104 GG genotype might have a higher risk of cervical cancer in the codominant (*p* = .045, OR = 1.90, 95% CI = 1.13–3.18) and recessive (*p* = .014, OR = 1.86, 95% CI = 1.12–3.09) models. We also analyzed the remaining SNPs (rs13281615, rs1562430, and rs2392780), and found no significant correlation.

**Table 3.** Genetic model analyses of five selected SNPs in *CASC21* and the risk of cervical cancer.

SNPs	Model	Genotype	Control	Case	OR (95% CI)	*p*
rs16902094	Codominant	A/A	288 (60.1%)	276 (55.9%)	1	**0.042**
		G/A	166 (34.7%)	172 (34.8%)	1.08 (.83–1.42)	
		G/G	25 (5.2%)	46 (9.3%)	**1.91 (1.14–3.20)**	
	Dominant	A/A	288 (60.1%)	276 (55.9%)	1	0.180
		G/A-G/G	191 (39.9%)	218 (44.1%)	1.19 (.92–1.54)	
	Recessive	A/A-G/A	454 (94.8%)	448 (90.7%)	1	**0.014**
		G/G	25 (5.2%)	46 (9.3%)	**1.86 (1.12–3.08)**	
	Log-additive	—	—	—	**1.24 (1.01–1.51)**	**0.039**
rs16902104	Codominant	C/C	287 (59.9%)	278 (56.3%)	1	**0.045**
		T/C	167 (34.9%)	170 (34.4%)	1.05 (.80–1.38)	
		T/T	25 (5.2%)	46 (9.3%)	**1.90 (1.13–3.18)**	
	Dominant	C/C	287 (59.9%)	278 (56.3%)	1	0.250
		T/C-T/T	192 (40.1%)	216 (43.7%)	1.16 (.90–1.50)	
	Recessive	C/C-T/C	454 (94.8%)	448 (90.7%)	1	**0.014**
		T/T	25 (5.2%)	46 (9.3%)	**1.86 (1.12–3.09)**	
	Log-additive	—	—	—	1.22 (.99–1.49)	0.056
rs13281615	Codominant	G/G	140 (29.3%)	136 (27.5%)	1	0.810
		A/G	220 (46%)	235 (47.6%)	1.10 (.82–1.49)	
		A/A	118 (24.7%)	123 (24.9%)	1.07 (.76–1.52)	
	Dominant	G/G	140 (29.3%)	136 (27.5%)	1	0.540
		A/G-A/A	338 (70.7%)	358 (72.5%)	1.09 (.83–1.44)	
	Recessive	G/G-A/G	360 (75.3%)	371 (75.1%)	1	0.960
		A/A	118 (24.7%)	123 (24.9%)	1.01 (.75–1.35)	
	Log-additive	—	—	—	1.04 (.87–1.23)	0.680
rs1562430	Codominant	T/T	326 (68.2%)	331 (67.1%)	1	0.920
		C/T	137 (28.7%)	147 (29.8%)	1.06 (.80–1.40)	
		C/C	15 (3.1%)	15 (3%)	.98 (.47–2.05)	
	Dominant	T/T	326 (68.2%)	331 (67.1%)	1	0.720
		C/T-C/C	152 (31.8%)	162 (32.9%)	1.05 (.80–1.38)	
	Recessive	T/T-C/T	463 (96.9%)	478 (97%)	1	0.930
		C/C	15 (3.1%)	15 (3%)	.97 (.47–2.00)	
	Log-additive	—	—	—	1.03 (.82–1.31)	0.780
rs2392780	Codominant	A/A	255 (53.2%)	267 (54%)	1	0.950
		G/A	195 (40.7%)	196 (39.7%)	.96 (.74–1.25)	
		G/G	29 (6%)	31 (6.3%)	1.02 (.60–1.75)	
	Dominant	A/A	255 (53.2%)	267 (54%)	1	0.820
		G/A-G/G	224 (46.8%)	227 (46%)	.97 (.75–1.25)	
	Recessive	A/A-G/A	450 (94%)	463 (93.7%)	1	0.890
		G/G	29 (6%)	31 (6.3%)	1.04 (.61–1.75)	
	Log-additive	—	—	—	.99 (.80–1.21)	0.900

SNP: Single nucleotide polymorphism; OR: Odds ratio; 95% CI: 95% confidence interval.

*p*-values were calculated by logistic regression analysis with adjustments for age, BMI, smoking, and drinking.

Bold indicates a statistically significant SNP (*p* < .05).

### 2.4. Stratified analysis of *CASC21* polymorphisms and the risk of cervical cancer

We stratified the cases and the control group by age, BMI, smoking, and drinking to eliminate the influence of confounding factors (Table 4). In the subgroup (age >51), rs16902094 (*p* = .018, OR = 2.43) and rs16902104 (*p* = .018, OR = 2.43) were found to be significantly correlated with the susceptibility of cervical cancer. Among subjects with BMI <24 kg/m^2^, rs16902094 (codominant: *p* = .043, OR = 2.16; and recessive: *p* = .014, OR = 2.23) and rs16902104 (codominant: *p* = .041, OR = 2.16; and recessive: *p* = .013, OR = 2.24) might confer to the risk-increasing effect on the occurrence of cervical cancer. Stratified analysis by smoking, rs16902094 and rs16902104 were related to the higher risk of cervical cancer in smokers under the codominant (*p* = .026, OR = 2.84; and *p* = .026, OR = 2.84), recessive (*p* = .012, OR = 2.65; and *p* = .012, OR = 2.65), and log-additive (*p* = .017, OR = 1.43; and *p* = .017, OR = 1.43) models, respectively. Moreover, we also observed the association of rs16902094 with the occurrence of cervical cancer (*p* = .042, OR = 2.06) in nondrinkers.

**Table 4.** Risk analysis of *CASC21* and cervical cancer in different genetic models according the stratification by age, BMI, smoking, and drinking.

SNP ID	Model	Genotype	OR (95%CI)	*p*	OR (95%CI)	*p*
Age stratification			Age ≤51 years		Age >51 years	
rs16902094	Codominant	A/A	1	0.450	1	0.056
		G/A	1.13 (0.76–1.69)		1.09 (0.75–1.59)	
		G/G	1.53 (0.76–3.08)		**2.51 (1.15–5.49)**	
	Dominant	A/A	1	0.330	1	0.250
		G/A-G/G	1.20 (0.83–1.74)		1.24 (0.86–1.77)	
	Recessive	A/A-G/A	1	0.280	1	**0.018**
		G/G	1.46 (0.74–2.90)		**2.43 (1.13–5.22)**	
	Log-additive	—	1.19 (0.89–1.59)	0.230	1.32 (0.98–1.76)	0.062
rs16902104	Codominant	C/C	1	0.480	1	0.059
		T/C	1.11 (0.75–1.66)		1.05 (0.72–1.53)	
		T/T	1.52 (0.75–3.06)		**2.48 (1.13–5.41)**	
	Dominant	C/C	1	0.380	1	0.330
		T/C-T/T	1.18 (0.81–1.71)		1.19 (0.83–1.71)	
	Recessive	C/C-T/C	1	0.280	1	**0.018**
		T/T	1.46 (0.74–2.90)		**2.43 (1.13–5.23)**	
	Log-additive	—	1.18 (0.89–1.57)	0.260	1.29 (0.96–1.72)	0.085
BMI stratification			BMI <24 kg/m^2^		BMI ≥24 kg/m^2^	
rs16902094	Codominant	A/A	1	**0.043**	1	0.380
		G/A	.91 (0.63–1.31)		1.23 (0.81–1.87)	
		G/G	**2.16 (1.10–4.21)**		1.64 (0.71–3.79)	
	Dominant	A/A	1	0.690	1	0.220
		G/A-G/G	1.07 (0.76–1.51)		1.28 (0.86–1.91)	
	Recessive	A/A-G/A	1	**0.014**	1	0.320
		G/G	**2.23 (1.16–4.30)**		1.51 (0.67–3.43)	
	Log-additive	—	1.20 (0.92–1.56)	0.180	1.26 (0.91–1.73)	0.160
rs16902104	Codominant	C/C	1	**0.041**	1	0.490
		T/C	0.90 (0.63–1.31)		1.15 (0.76–1.75)	
		T/T	**2.16 (1.11–4.22)**		1.60 (0.69–3.69)	
	Dominant	C/C	1	0.710	1	0.350
		T/C-T/T	1.07 (0.76–1.50)		1.21 (0.81–1.80)	
	Recessive	C/C-T/C	1	**0.013**	1	0.320
		T/T	**2.24 (1.16–4.32)**		1.51 (0.67–3.43)	
	Log-additive	—	1.20 (0.92–1.56)	0.190	1.21 (0.88–1.67)	0.250
Smoking stratification			Smokers		Non-smokers	
rs16902094	Codominant	A/A	1	**0.026**	1	0.560
		G/A	1.22 (0.82–1.80)		0.99 (0.67–1.44)	
		G/G	**2.84 (1.27–6.38)**		1.43 (0.72–2.83)	
	Dominant	A/A	1	0.082	1	0.780
		G/A-G/G	1.39 (0.96–2.01)		1.05 (0.74–1.51)	
	Recessive	A/A-G/A	1	**0.012**	1	0.290
		G/G	**2.65 (1.20–5.87)**		1.43 (0.74–2.80)	
	Log-additive	—	**1.43 (1.06–1.93)**	**0.017**	1.10 (0.83–1.45)	0.500
rs16902104	Codominant	C/C	1	**0.026**	1	0.510
		T/C	1.21 (0.82–1.79)		0.93 (0.64–1.36)	
		T/T	**2.84 (1.27–6.37)**		1.42 (0.72–2.81)	
	Dominant	C/C	1	0.084	1	0.970
		T/C-T/T	1.38 (0.96–2.00)		1.01 (0.70–1.44)	
	Recessive	C/C-T/C	1	**0.012**	1	0.270
		T/T	**2.65 (1.20–5.87)**		1.46 (0.75–2.84)	
	Log-additive	—	**1.43 (1.06–1.92)**	**0.017**	1.07 (0.81–1.42)	0.620
Drinking stratification			Drinkers		Non-drinkers	
rs16902094	Codominant	A/A	1	0.290	1	0.110
		G/A	1.09 (0.74–1.60)		1.09 (0.74–1.59)	
		G/G	1.79 (0.86–3.72)		**2.13 (1.03–4.41)**	
	Dominant	A/A	1	0.360	1	0.280
		G/A-G/G	1.18 (0.82–1.70)		1.22 (0.85–1.75)	
	Recessive	A/A-G/A	1	0.130	1	**0.042**
		G/G	1.73 (0.84–3.55)		**2.06 (1.01–4.20)**	
	Log-additive	—	1.22 (0.91–1.62)	0.180	1.28 (0.96–1.70)	0.092

SNP: Single nucleotide polymorphism; OR: Odds ratio; 95% CI: 95% confidence interval.

*p*-values were calculated by logistic regression analysis with adjustments for age, BMI, smoking, and drinking.

Bold indicates a statistically significant SNP (*p* < .05).

Furthermore, genetic model analyses for five selected SNPs in *CASC21* and the risk of cervical squamous cell carcinoma were performed, and the results were shown in Table 5. Rs16902094 (*p* = .024, OR = 1.39) and rs16902104 (*p* = .041, OR = 1.35) were correlated with the increased susceptibility of cervical squamous cell carcinoma.

**Table 5.** Genetic model analyses for five selected SNPs in *CASC21* and the risk of cervical squamous cell carcinoma.

SNP ID	Model	Genotype	Control	Case	OR (95% CI)	*p*
rs16902094	Codominant	A/A	288 (60.1%)	88 (51.5%)	1	0.074
		G/A	166 (34.7%)	68 (39.8%)	1.34 (0.92–1.94)	
		G/G	25 (5.2%)	15 (8.8%)	**2.05 (1.03–4.08)**	
	Dominant	A/A	288 (60.1%)	88 (51.5%)	1	0.051
		G/A-G/G	191 (39.9%)	83 (48.5%)	1.43 (1.00–2.03)	
	Recessive	A/A-G/A	454 (94.8%)	156 (91.2%)	1	0.089
		G/G	25 (5.2%)	15 (8.8%)	1.82 (0.93–3.56)	
	Log-additive	—	—	—	**1.39 (1.05–1.84)**	**0.024**
rs16902104	Codominant	C/C	287 (59.9%)	90 (52.6%)	1	0.110
		T/C	167 (34.9%)	66 (38.6%)	1.27 (0.87–1.84)	
		T/T	25 (5.2%)	15 (8.8%)	2.00 (1.00–3.99)	
	Dominant	C/C	287 (59.9%)	90 (52.6%)	1	0.091
		T/C-T/T	192 (40.1%)	81 (47.4%)	1.36 (0.95–1.94)	
	Recessive	C/C-T/C	454 (94.8%)	156 (91.2%)	1	0.089
		T/T	25 (5.2%)	15 (8.8%)	1.82 (0.93–3.56)	
	Log-additive	—	—	—	**1.35 (1.01–1.78)**	**0.041**

SNP: Single nucleotide polymorphism; OR: Odds ratio; 95% CI: 95% confidence interval.

*p*-values were calculated by logistic regression analysis with adjustments for age, BMI, smoking, and drinking.

Bold indicates a statistically significant SNP (*p* < .05).

There was no significant difference between the remaining SNPs and cervical cancer in the stratification analysis (data not shown).

### 2.5. FPRP analysis

False positive reporting probability (FPRP) analysis (Table 6) exhibited the positive results of rs16902094 and rs16902104 for cervical cancer susceptibility in the overall analysis with .1 prior probability level and FPRP < .2. The effects of rs16902094 and rs16902104 on cervical cancer risk in smokers. In addition, rs16902094 was also significantly associated with the risk of cervical squamous cell carcinoma with an FPRP value of < .2 despite a prior probability level of .1.

**Table 6.** False-positive report probability for the associations of *CASC21* variants with the risk of cervical cancer.

SNP ID	Model	OR (95% CI)	Prior probability				
			0.25	0.1	0.01	0.001	0.0001
Overall							
rs16902094	Codominant	1.91 (1.14–3.20)	**0.069**	**0.181**	0.709	0.961	0.996
	Recessive	1.86 (1.12–3.08)	**0.072**	**0.190**	0.720	0.963	0.996
	Log-additive	1.24 (1.01–1.51)	**0.088**	0.225	0.762	0.970	0.997
rs16902104	Codominant	1.90 (1.13–3.18)	**0.070**	**0.185**	0.714	0.962	0.996
	Recessive	1.86 (1.12–3.09)	**0.075**	**0.196**	0.729	0.964	0.996
Age >51 years							
rs16902094	Codominant	2.51 (1.15–5.49)	**0.182**	0.401	0.880	0.987	0.999
	Recessive	2.43 (1.13–5.22)	**0.182**	0.400	0.880	0.987	0.999
rs16902104	Codominant	2.48 (1.13–5.41)	**0.186**	0.407	0.883	0.987	0.999
	Recessive	2.43 (1.13–5.23)	**0.184**	0.403	0.881	0.987	0.999
BMI <24 kg/m^2^							
rs16902094	Codominant	2.16 (1.10–4.21)	**0.148**	0.342	0.851	0.983	0.998
	Recessive	2.23 (1.16–4.30)	**0.118**	0.287	0.816	0.978	0.998
rs16902104	Codominant	2.16 (1.11–4.22)	**0.150**	0.347	0.854	0.983	0.998
	Recessive	2.24 (1.16–4.32)	**0.116**	0.283	0.813	0.978	0.998
Smokers							
rs16902094	Codominant	2.84 (1.27–6.38)	**0.148**	0.343	0.852	0.983	0.998
	Recessive	2.65 (1.20–5.87)	**0.167**	0.376	0.869	0.985	0.999
	Log-additive	1.43 (1.06–1.93)	**0.056**	**0.150**	0.661	0.952	0.995
rs16902104	Codominant	2.84 (1.27–6.37)	**0.147**	0.340	0.850	0.983	0.998
	Recessive	2.65 (1.20–5.87)	**0.167**	0.376	0.869	0.985	0.999
	Log-additive	1.43 (1.06–1.92)	**0.050**	**0.137**	0.635	0.946	0.994
Non-drinkers							
rs16902094	Codominant	2.13 (1.03–4.41)	0.224	0.465	0.905	0.990	0.999
	Recessive	2.06 (1.01–4.20)	0.231	0.474	0.908	0.990	0.999
cervical squamous cell carcinoma							
rs16902094	Codominant	2.05 (1.03–4.08)	0.206	0.438	0.896	0.989	0.999
	Log-additive	1.39 (1.05–1.84)	**0.061**	**0.162**	0.680	0.955	0.995
rs16902104	Log-additive	1.35 (1.01–1.78)	**0.091**	0.232	0.768	0.971	0.997

SNP: Single nucleotide polymorphism; OR: Odds ratio; 95% CI: 95% confidence interval.

The false-positive report probability threshold level was set at .2, and Bold represents that noteworthy findings are presented.

### 2.6. The association between *CASC21* haplotypes and the risk of cervical cancer

**Figure 1.**
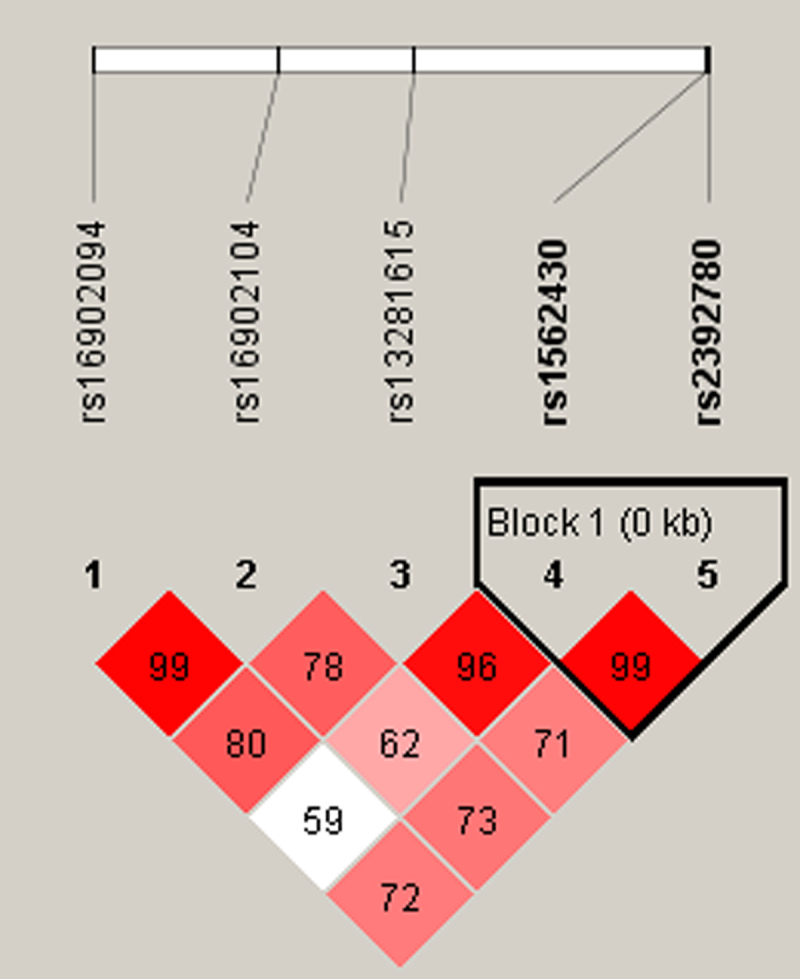
LD plots of five SNPs in the *CASC21* gene.

Moreover, haplotype analysis was performed to estimate the association between *CASC21* haplotypes and the risk of cervical cancer. As shown in Figure 1, rs1562430 and rs2392780 are in linkage disequilibrium. The haplotype frequency distribution was shown in Table 7. The association between the *CASC21* haplotype and cervical cancer susceptibility was investigated; however, there was no significant relationship between these haplotypes and cervical cancer risk (*p* > .05).

**Table 7.** Haplotype analysis for the effect of *CASC21* haplotypes on the risk of cervical cancer.

SNPs ID	Haplotype	Frequency		Crude analysis		Adjusted by age	
		Control	Case	OR (95% CI)	*p*	OR (95% CI)	*p*
rs1562430|rs2392780	TA	0.735	0.739	1		1	
rs1562430|rs2392780	CG	0.173	0.180	1.03 (0.81–1.31)	0.800	1.03 (0.81–1.31)	0.800
rs1562430|rs2392780	TG	0.091	0.081	0.90 (0.65–1.23)	0.500	0.90 (0.66–1.24)	0.520

*CASC21* block comprises the two closely linked SNPs (rs1562430 and rs2392780).

SNP:Single nucleotide polymorphism; OR: Odds ratio; 95% CI: 95% confidence interval.

p-values were calculated by logistic regression analysis with adjustments for age, BMI, smoking, and drinking.

### 2.7. SNP – SNP interaction analysis using MDR

**Figure 2.**
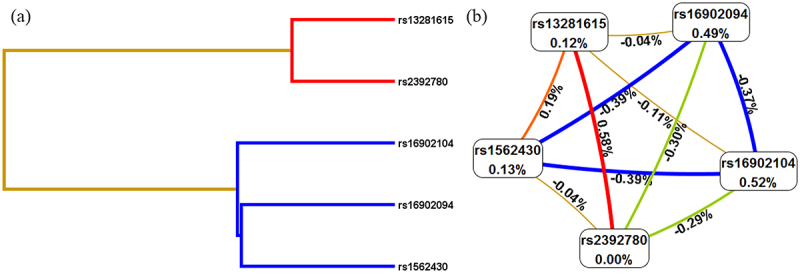
SNP-SNP interaction dendrogram (a) and Fruchterman-Reingold (b).

Multifactor dimensionality reduction (MDR)was used to analyze the SNP – SNP interaction between these five SNPs (rs16902094, rs16902104, rs13281615, rs1562430, and rs2392780) in the occurrence of cervical cancer. As shown in Table 8, the best model was the combination of rs13281615 and rs2392780 (Bal. Acc. CV testing = .5334, CV consistency = 10/10. *p* = .0022). The Dendogram plot in Figure 1(a) and the Fruchterman Rheingold plot in Figure 2(b) represented the interaction between SNPs. The red color in Figure 2(a) indicated that there is a synergistic effect between the two SNPs, while the blue color indicates a negative correlation between the two SNPs. The entropy interaction graphical model (Figure 2(b)) revealed that rs13281615 and rs2392780 had significant synergistic interaction (.58%) sharing the positive information gain concerning cervical cancer, whereas rs16902094 (.49%) and rs16902104 (.52%) were the main influential attribution factor for cervical cancer risk.

**Table 8.** Summary of SNP – SNP interactions on the risk of cervical cancer analyzed by MDR method.

Model	Bal. Acc. CV Training	Bal. Acc. CV Testing	CV Consistency	OR (95% CI)	*p*
rs16902094	0.5230	0.5010	6/10	**1.88 (1.13–3.12)**	**0.0130**
rs13281615, rs2392780	0.5495	0.5334	10/10	**1.49 (1.15–1.93)**	**0.0022**
rs16902094, rs13281615, rs2392780	0.561	0.5115	9/10	**1.65 (1.27–2.15)**	**0.0002**
rs16902094, rs13281615, rs1562430, rs2392780	0.5659	0.5073	6/10	**1.66 (1.28–2.16)**	**0.0001**
rs16902094, rs16902104, rs13281615, rs1562430, rs2392780	0.5694	0.5125	10/10	**1.76 (1.35–2.29)**	**<.0001**

SNP: Single nucleotide polymorphism; OR: Odds ratio; 95% CI: 95% confidence interval.

p-values were calculated by χ2.

Bold indicates a statistically significant SNP (p < .05).

## 3. Discussion

In our study, we selected five SNPs on the *CASC21* gene (rs16902094, rs16902104, rs13281615, rs1562430, and rs2392780) to explore the correlation between these polymorphisms and the risk of cervical cancer. Our results suggested that two SNPs (rs16902094, rs16902104) might contribute to the increased risk of cervical cancer in the Chinese Han population, especially in the subjects aged >51 years, population with BMI <24 kg/m^2^, smokers, and patients with cervical squamous cell carcinoma. Moreover, we also observed the association of rs16902094 with the occurrence of cervical cancer in nondrinkers. These results firstly found that the genetic polymorphism of *CASC21* might play an important role in the occurrence of cervical cancer in the Han population from northwest China, which increases the understanding of the role of *CASC21* in cervical carcinogenesis.

*CASC21* might promote cell proliferation, regulate cell cycle, and enhance tumor metastasis in colon cancer ^18,19^. *CASC21* promotes the growth of cancer cells by regulating cyclin-dependent kinase 6(CDK6) ^19^. Downregulation of CDK6 can inhibit the proliferation ability of cervical cancer and promote the apoptosis of cervical cancer cells.^20^ Little has been reported about the contribution of *CASC21* variants to the susceptibility of tumors. This study is the first to show that two SNPs (rs16902094, rs16902104) might contribute to the increased risk of cervical cancer in the Chinese Han population. Rs16902094, located in the intron region was associated with susceptibility to prostate cancer in several European populations.^15^ Rs16902094 and rs16902104 are adjacent to each other, both of which are on the *CASC21* gene. According to our experimental results, our results firstly suggested that rs16902094, and rs16902104 might contribute to the increased risk of cervical cancer in the Chinese Han population. MDR analysis can be speculated that rs16902094 (.49%) and rs16902104 (.52%) were the main influential attribution factor for cervical cancer risk. Bioinformatics analysis suggested that the possible function of rs16902094 and rs16902104 might be related to promoter/enhancer histone marks, DNAse, motifs changed, NHGRI/EBI GWAS hits, and selected eQTL hits. This suggests that these loci may play a part in cervical cancer development by influencing *CASC21* gene expression. However, this hypothesis requires to be explored by further functional research.

It is well known that genetic, environmental, and behavioral risk factors may affect cervical cancer development. Katrina V Fox, etc., the study also found that the risk of cervical cancer increases with age.^21^ In the age stratification of more than 51 years old, the risk of disease of rs16902094 and rs16902104 has been significantly increased, which may partly reflect the age – gene interactions in the occurrence of cervical cancer.

Increased BMI has been considered to increase the risk of many cancers, including cervical cancer.^22^ Underweight women had significantly lower cervical cancer screening rates compared to other BMI categories.^23^ Both extremes of weight (underweight and overweight/obesity) were associated with worse survival in patients with cervical cancer.^24^ Moreover, the lower risk of cervical precancer and higher risk of cervical cancer with increasing body mass index were observed.^25^ Interestingly, rs16902094 and rs16902104 might confer the risk-increasing effect on the occurrence of cervical cancer among subjects with BMI <24 kg/m^2^. Based on these results, we speculated the age and BMI for the risk association of *CASC21* polymorphisms with cervical cancer susceptibility.

Tobacco smoking is an important risk factor for cervical neoplasia. Smoking status, duration, and intensity are related to a twofold increased risk of high-grade cervical dysplasia and invasive carcinoma.^26^ Our finding displayed that rs16902094 and rs16902104 were related to the higher risk of cervical cancer in smokers. Alcohol abuse can decrease pelvic control and survival in cervical cancer and increase the risk of cervical cancer.^27^ Moreover, we also observed the association of rs16902094 with the occurrence of cervical cancer in nondrinkers. Therefore, the role of smoking, drinking, and heredity in the occurrence of cervical cancer needs to be confirmed in further studies.

There are still some limitations in our study, due to the sample size and race. The results only apply to the Han nationality in northwest China. We will continue to study the impact on other ethnic groups. Besides, due to insufficient collection of patients’ HPV information, the correlation between *CASC21* and HIV infection in cervical carcinogenesis needs to be further studied.

## 4. Materials and methods

### 4.1. Ethics statement

We followed the Helsinki Declaration of the World Medical Association and subsequent amendments. This study has been approved by the Ethics Committee of the People’s Hospital of Xinjiang Uygur Autonomous Region (Approval Document No: KY2020041053). All participants in this study were informed and signed informed consent forms.

### 4.2. Subjects

In order to ensure the accuracy and credibility of the research results, before we plan to conduct this study, we used G*power 3.1.9.7 software (https://stats.idre.ucla.edu/other/gpower/) to estimate the sample size of the case group and the control group through the independent sample T-test. The specific parameters we set are as follows: Tail=two, effect size d = .2; α error probability = .05; power (1-β err prob) = .85, allocation ratio N2/N1 = 1. This calculation yielded a sample consisting of at least 450 cases and 450 controls. In this study, a total of 973 participants within 494 cervical cancer cases and 479 healthy controls were recruited from People’s Hospital of Xinjiang Uygur Autonomous Region from April 2020 to April 2022, which is larger than the total sample size recommended by G*power and statistic power > 85%. In this study, 494 Han nationalities in northwest China unrelated blood samples were randomly collected. According to the diagnostic criteria,^28^ all the patients were determined to have cervical cancer. The samples were collected from the People’s Hospital of Xinjiang Uygur Autonomous Region. All the patients have no history of radiotherapy or chemotherapy. In addition, 479 control samples were selected from the Han nationality in northwest China. All controls were confirmed by the pathology department to have negative cervical cytology and had no history of cancer, infection, or acute/chronic lesions. Demographic and clinical information were collected from the standardized questionnaires and medical records.

### 4.3. Variant selection and genotyping

We randomly selected rs16902094, rs16902104, rs13281615, rs1562430, and rs2392780 loci in *CASC21* gene for genotyping based on the following: 1) MAF > .05 in the Chinese Han population from 1000 Genomes Chinese Han Beijing population and dbSNP database; 2) HWE > .05, and the call rate for genotyping > 99.5%; 3) the related literature of *CASC21* polymorphisms associated with the risk of prostate and breast cancers,^15–17^ but no reports about cervical cancer. HaploReg v4.1 (https://pubs.broadinstitute.org/mammals/haploreg/haploreg.php) were used for the potential function of these polymorphisms.

Peripheral blood samples (5 mL) were collected in EDTA-coated tubes. The GoldMag DNA Purification Kit (GoldMag Co. Ltd., Xi’an, China) was used to extract genomic DNA, which was quantified by NanoDrop 2000 (Thermo Scientific, Waltham, MA, USA) and stored at −20°C. The MassARRAY platform is based on MALDI-TOF (matrix-assisted laser desorption/ionization – time of flight) mass spectrometry.^29,30^ The analytical accuracy of MALDI-TOF MS is quite high, .1–.01% of the determined mass. Agena MassARRAY system (Agena, San Diego, CA, U.S.A.) was used for SNPs genotyping. The specific steps included: PCR amplification of the target sequence, and mass spectrometry to distinguish nucleic acid molecules of different molecular weights. The primer-related information was shown in Table S1. In addition, this study also set up double wells for each sample to ensure the accuracy of the results. About 10% of the samples were randomly selected and re-genotyped for quality control, and the concordance rate was 100%.

### 4.4. Statistical analyses

PLINK software was used to perform statistical analysis on the original data. The chi-square test compared the differences in SNP genotypes between the case and control groups, and *p* < .05 indicated that the locus might have a significant correlation with the risk of cervical cancer. HWE test was performed on the control group, and *p* > .05 indicated that the population was genetically balanced, and the survey data was reliable. OR and 95% CI adjusted by age, BMI, smoking, and drinking were used to evaluate the influence of *CASC21* variants on the risk of cervical cancer under the different models (codominant, dominant, recessive, and log‐additive).^31^ FPRP analysis was used to evaluate the noteworthy associations of the significant findings. The FPRP threshold is .2, and the prior probability is .1, which is used to evaluate the significant association of significant findings. D’ values for pairwise linkage disequilibrium (LD) plots were generated by Haploview software (version 4.2), and the correlation of *CASC21* haplotypes with cervical cancer risk was evaluated by logistic regression model. MDR is specifically designed to identify correlations between increased risk and genetic variation for complex diseases in humans. MDR version 3..2 was applied to explore the association between the risk of cervical cancer and multi-SNP interactions. Cross-validation can reduce false positive results caused by random grouping of data to a certain extent, which is usually used to assess the statistical significance of MDR models.

## 5. Conclusion

In our study, we found that both rs16902094 and rs16902104 polymorphisms increase the risk of cervical cancer in the Han population from northwest China. Our findings add to our knowledge regarding the effect of *CASC21* on cervical carcinogenesis.

## Supplementary Material

supplement table.docx

## Data Availability

The datasets used and analyzed in the current study are available from the corresponding author upon reasonable request.
