# Supplementary material for: The relationship between long non-coding gene CASC21 polymorphisms and cervical cancer
Source: Cancer Biol Ther. 2024 Mar 11;25(1):2322207. doi: 10.1080/15384047.2024.2322207 (PMC10936591; doi:10.1080/15384047.2024.2322207)
Supplement: supplement table.docx [file KCBT_A_2322207_SM8196.docx]

Table S1 Primers used for this study

| SNP_ID | 2nd - PCRP | 1st - PCRP | UEP DIR | UEP SEQ |
| --- | --- | --- | --- | --- |
| rs16902094 | ACGTTGGATGATCAGCTATCTCAGTCTCAC | ACGTTGGATGCTGGATTAGATACAGGCCAC | R | gtcaACAATAACCCAAATGGACT |
| rs16902104 | ACGTTGGATGCAGTTTACACCACTTGACCC | ACGTTGGATGAGGATTGAAAGAGGAGGAG | F | GACTTAAGATACAAACCTCC |
| rs13281615 | ACGTTGGATGAACCCCCTACTCAGAATATC | ACGTTGGATGCCTGGAATCTAGGGATGTAG | R | ggccGAATATCTGCGTTCTGC |
| rs1562430 | ACGTTGGATGCGAAGTGCATAGACAACTCC | ACGTTGGATGTCCTCTTCACATATCTGCCC | F | cCAGCTACTTGAGCTCCTAAATATC |
| rs2392780 | ACGTTGGATGCATGCATCAGAGTGTTAGAC | ACGTTGGATGTTTGCCTGTCTTTGGTTAGC | F | GACAATAAGATGCAGTGTAAA |

PCR: polymerase chain reaction, UEP: unextended mini-sequencing primer.
